# Characterization of Uranyl (UO_2_^2+^) Ion Binding to Amyloid Beta (Aβ) Peptides: Effects on Aβ Structure and Aggregation

**DOI:** 10.1021/acschemneuro.3c00130

**Published:** 2023-07-24

**Authors:** Elina Berntsson, Faraz Vosough, Andra Noormägi, Kärt Padari, Fanny Asplund, Maciej Gielnik, Suman Paul, Jüri Jarvet, Vello Tõugu, Per M. Roos, Maciej Kozak, Astrid Gräslund, Andreas Barth, Margus Pooga, Peep Palumaa, Sebastian K. T. S. Wärmländer

**Affiliations:** † Chemistry Section, Arrhenius Laboratories, Stockholm University, 106 91 Stockholm, Sweden; ‡ Department of Chemistry and Biotechnology, Tallinn University of Technology, 19086 Tallinn, Estonia; § Institute of Molecular and Cell Biology, University of Tartu, 50090 Tartu, Estonia; ∥ Department of Molecular Biology and Genetics, Aarhus University, 8000 Aarhus, Denmark; ⊥ CellPept Sweden AB, Kvarngatan 10B, 118 47 Stockholm, Sweden; # Institute of Environmental Medicine, Karolinska Institutet, 171 77 Stockholm, Sweden; ¶ University Healthcare Unit of Capio St. Göran Hospital, 112 81 Stockholm, Sweden; ∇ Institute of Technology, University of Tartu, 50090 Tartu, Estonia; ○ Department of Biomedical Physics, Institute of Physics, Faculty of Physics, Adam Mickiewicz University, 61-712 Poznań, Poland; ⧫ SOLARIS National Synchrotron Radiation Centre, Jagiellonian University, 31-007 Kraków, Poland

**Keywords:** Alzheimer’s disease, amyloid aggregation, metal−protein binding, neurodegeneration, heavy metal toxicity

## Abstract

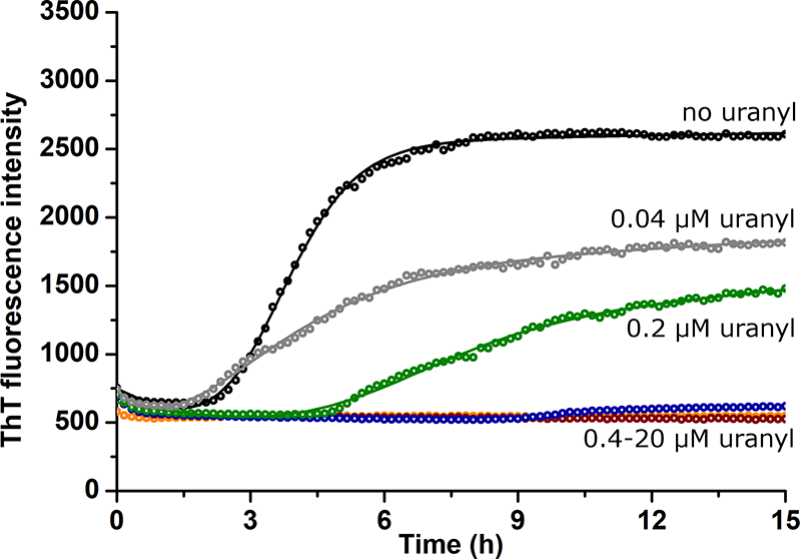

Uranium (U) is naturally present in ambient air, water, and soil, and depleted uranium (DU) is released into the environment via industrial and military activities. While the radiological damage from U is rather well understood, less is known about the chemical damage mechanisms, which dominate in DU. Heavy metal exposure is associated with numerous health conditions, including Alzheimer’s disease (AD), the most prevalent age-related cause of dementia. The pathological hallmark of AD is the deposition of amyloid plaques, consisting mainly of amyloid-β (Aβ) peptides aggregated into amyloid fibrils in the brain. However, the toxic species in AD are likely oligomeric Aβ aggregates. Exposure to heavy metals such as Cd, Hg, Mn, and Pb is known to increase Aβ production, and these metals bind to Aβ peptides and modulate their aggregation. The possible effects of U in AD pathology have been sparsely studied. Here, we use biophysical techniques to study in vitro interactions between Aβ peptides and uranyl ions, UO_2_^2+^, of DU. We show for the first time that uranyl ions bind to Aβ peptides with affinities in the micromolar range, induce structural changes in Aβ monomers and oligomers, and inhibit Aβ fibrillization. This suggests a possible link between AD and U exposure, which could be further explored by cell, animal, and epidemiological studies. General toxic mechanisms of uranyl ions could be modulation of protein folding, misfolding, and aggregation.

## 1 Introduction

Alzheimer’s disease (AD) is the most common neurodegenerative disease among elderly people.^1,2^ The main AD risk factors are old age and genetic factors such as unfavorable alleles of the ApoE gene and sometimes Down’s syndrome^1,2^ and also environmental risk factors such as smoking, diabetes, and traumatic brain injury.^2−4^ Identifying molecular targets to diagnose and treat the disease is imperative,^5,6^ and a number of drug candidates have been proposed with varying success.^7−10^

The main molecular events underlying AD pathology appear to be the aggregation of intrinsically disordered amyloid-β (Aβ) peptides and tau proteins into toxic soluble oligomers^11−13^ and then into insoluble amyloid fibrils or tangles^14^ that deposit as plaques in the brains of AD patients.^2,15^ The Aβ peptides are cleaved by β- and γ-secretase enzymes from the Aβ precursor protein (APP) into peptides of different lengths,^16^ where Aβ (1–40), i.e., Aβ_40_, and Aβ (1–42), i.e., Aβ_42_, are the most common.^17^ Both variants are unstructured monomers in an aqueous solution but can adopt β-sheet^18^ or α-helix^19^ secondary structures in other environments.^20^ The β-sheet structure is of particular interest as Aβ peptides in β-sheet hairpin conformations appear to be the building blocks of the aggregated fibrils.^18^

The accumulation and aggregation of Aβ peptides can be influenced by a number of interacting factors,^3,21^ such as other proteins and peptides,^10,22,23^ including other amyloid peptides/proteins,^24−27^ cationic molecules and metal ions,^28−30^ and various small molecules.^8^ Redox-active metal ions such as Cu(II) and Fe(II) are of special interest^29,31,32^ as they are present in the plaques of AD brains.^33,34^ For example, they can generate harmful oxygen radicals (reactive oxygen species, ROS) that may contribute to the AD pathology.^35−38^ Heavy metals are generally known to be toxic, but their toxic mechanisms are not fully understood.^39^ Known molecular mechanisms for metal toxicity include molecular and ionic mimicry,^40^ but other mechanisms are also possible, such as modulation of protein misfolding or aggregation.^41−43^ Thus, lead (Pb) ions not only compete with ions of essential metals such as zinc and calcium,^44^ but they also increase the expression of APP and β-secretase,^45^ and Pb(IV) ions bind to Aβ peptides and modulate their aggregation.^3^ Similar effects on Aβ production and aggregation have been observed for the heavy metals mercury and cadmium.^3,31,45−48^

Uranium (U) is a heavy metal with known neurotoxic effects,^39^ but these effects are sparsely studied, and a relation between U exposure and AD has not been established.^49^ However, the blood–brain barrier does not prevent the transfer of U into the central nervous system (CNS),^50^ and U has been shown to accumulate in the brain.^51^ Uranium has no biological function in the human body,^52,53^ and the adverse health effects of U exposure involve combined chemical and radiological mechanisms.^54,55^ Although the chemical toxicity is more severe,^56^ the radiological damage mechanisms are currently better understood.^39,55^ The chemical toxicity obviously dominates in depleted uranium (DU), where the amount of the ^235^U isotope has been significantly reduced in favor of the less radioactive (i.e., longer half-life) ^238^U isotope.^52^ As DU is used in military equipment, including certain ammunitions,^57^ DU contamination has emerged as a potential environmental problem in regions of war such as Iraq and the Balkans,^58−64^ with unclear health consequences for soldiers and civilians.^52,54,65−67^ To what extent DU contributes to leukemia or lung cancer remains debated.^68−71^

Here, we use biophysical spectroscopic and imaging techniques to investigate the binding interactions between uranyl ions and the three Aβ peptide variants Aβ_40_, Aβ_42_, and Aβ_40_ (H6A, H13A, and H14A) mutant. Of particular interest is the effect of uranyl on the Aβ structure and aggregation.

### 1.1 Chemical, Environmental, and Toxicological Aspects of Uranium and Uranyl Ions

Uranium is present in ambient air, water, and soil. The highest human exposures result from drinking well water in geological regions rich in U.^39,53,72−75^ Regulatory agencies have limited the highest allowed concentration of U in drinking water to 30 μg/L,^76^ replacing a previous WHO limit of 15 μg/L. Absorption of U is low regardless of exposure route and highly dependent on its solubility.^39^ Inhaled U-dust particles of low solubility can be retained in tissues for many years.^77^ Occupational exposure to U has historically involved workers in the production of phosphate fertilizers, workers producing glazed pottery, and miners handling uranium oxide, so-called “yellowcake”.^39,78^ Sleep disturbances and possibly encephalitis have been linked to U exposure in former U mining districts in Kazakhstan.^79^

The main target for U toxicity is the kidney where atrophy and necrosis of glomerular walls have been noted.^39,80−82^ Uranium also accumulates in bone.^39,60,83−85^ Uranium crosses the blood–brain barrier to accumulate in the CNS.^50^ Here, inhalation and ingestion of U yields heterogeneous but specific accumulations, most prominent in the hippocampus region,^86^ responsible for memory recall. Rats surgically implanted with U pellets for 6 months have shown the presence of U in the cortex, midbrain, and cerebellum.^87^ A study on mice showed toxic effects of DU in the mouse fetus,^88^ indicating that U can also cross the placental barrier.

There are currently no known correlations between uranium exposure and diseases such as leukemia,^73^ stomach cancer,^89^ liver or bladder cancer,^90^ or, as mentioned above, AD.^49^ One study, however, found cerebrospinal fluid U concentrations, albeit at low overall concentrations, to be significantly elevated in 17 patients with amyotrophic lateral sclerosis (ALS) when compared to 10 controls.^91^ Studies in rodents have related U exposure to distorted social behavior^92^ and weakened sensorimotor behavior.^93^ Laboratory experiments using organisms such as rats and *Caenorhabditis elegans* have concluded with a low acute neurotoxic potential of U following exposure and a protective potential from the small metal-regulating protein metallothionein.^87,94^ Protective effects have also been observed for the ghrelin hormone^95^ and for antioxidant agents, including glutathione.^96,97^

The most predominant, most stable, and most relevant form of uranium in aerobic environments is the uranyl oxycation, UO_2_^2+^. This ion is common in uranium-containing minerals, but it is also water-soluble and is present in the ocean at a concentration of 13.7 nM.^98^ The uranyl ion is paramagnetic,^99,100^ and in aqueous solution, it acts as a weak acid with a p*K*_a_ of around 4.2.^101^ It behaves as a hard acceptor and prefers to form complexes with fluoride and oxygen donor atoms, preferably in planar geometry involving four, five, or even six binding ligands.^102^ The capacity to accommodate five or six equatorial ligands in pentagonal or hexagonal bipyramidal coordination separates the uranyl ion from most other metal ions.^98^ Thus, these uncommon binding geometries have been employed in attempts to design uranyl-specific binding proteins, e.g., to extract uranium from seawater.^98^ As the most common form of uranium, most experimental studies of uranium toxicity have actually been conducted with uranyl ions rather than with metallic uranium, which is extremely rare in nature.

Interestingly, U intoxication and AD appear to have a common risk factor in the gene coding for apolipoprotein E (ApoE). U exposure in mice was found to induce cognitive impairment in ApoE-deficient (ApoE–/−) males, together with some changes in cholesterol levels and metabolism.^103,104^ This is consistent with the ApoEε4 allele of the ApoE gene being a risk factor for mercury toxicity^105,106^ and with ApoE-deficient mice showing increased iron accumulation in tissue over time.^107^ It therefore appears likely that the ApoE protein is involved in regulating metal homeostasis. The ApoEε4 allele is further linked to an increased probability of developing AD.^108−112^ Why the ApoE gene is a risk factor for both heavy metal toxicity and AD is currently unclear, and various explanations have been proposed.^108,113,114^ The previously suggested hypothesis that ApoE might bind and transport metal ions via Cys residues,^105,106^ which are present in ApoE2 and ApoE3 but not in ApoE4, has recently been called into question.^115^

## 2 Results

### 2.1 ThT Fluorescence Measurements of Aβ_40_ Aggregation Kinetics

**Figure 1:**
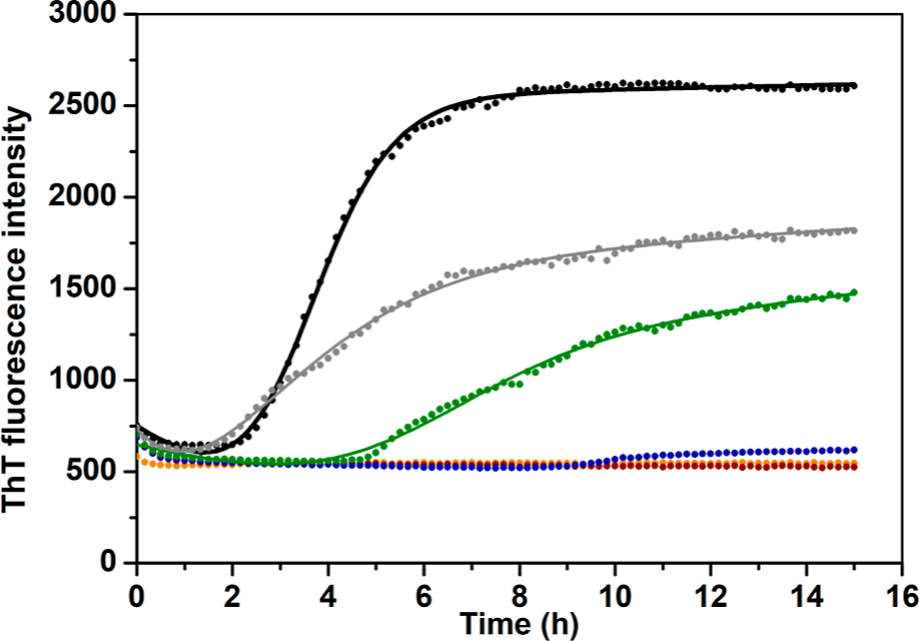
Monitoring the fibril formation of Aβ_40_ peptides via thioflavin T fluorescence. 20 μM Aβ_40_ was incubated at 37 °C in 20 mM MES buffer, pH 7.3, together with different concentrations of uranyl acetate: 0 μM (black); 0.04 μM (gray); 0.2 μM (green); 0.4 μM (blue); 2 μM (red); and 20 μM (orange).

The fluorescence intensity of ThT, a common marker for amyloid material,^116^ was monitored when samples of 20 μM Aβ_40_ peptides in 20 mM MES buffer, pH 7.3, were incubated with shaking for 15 h at 37 °C, together with different concentrations (0; 0.04; 0.2; 0.4; 2; and 20 μM) of uranyl acetate (Figure 1).

Fitting eq 1 to the ThT fluorescence curves in Figure 1 produced the kinetic parameters *T*_lag_ and *t*_1/2_ (Table 1). For the three highest concentrations of uranyl acetate, i.e., 0.4, 2, and 20 μM, the ThT kinetic curves are mostly flat, and fitting eq 1 to these curves was not possible. The flat shape of the three curves indicates that no or very little amyloid material has formed, although for the 0.4 μM uranyl sample, small amounts of the ThT-binding material have begun to form after approximately 9 h. It is obvious that the amount of the amyloid material formed at the end of the measurements, i.e., ΔThT, strictly decreases with the amount of added uranyl acetate (Figure 1 and Table 1). The two samples with 0 and 0.04 μM uranyl both have aggregation half-times of around 3 h and lag times of around 2 h under the experimental conditions (Table 1). The aggregation of the 0.2 μM uranyl sample is clearly slower, with a half-time of almost 7 h and a lag time of around 5 h. Given the visually estimated lag time of around 9 h for the 0.4 μM uranyl sample and that the 2 and 20 μM uranyl samples show no obvious signs of aggregation after 15 h, uranyl ions clearly increase the lag time for Aβ_40_ aggregation.

**Table 1:** Kinetic Parameters for Aβ_40_ Aggregation in the Presence of Different Concentrations of Uranyl Acetate, Derived from the ThT Fluorescence Curves Shown in Figure 1^a^

uranyl ions	0 μM	0.04 μM	0.2 μM	0.4 μM	2 μM	20 μM
*t*_1/2_ (h)	3.5 ± 0.3	3.1. ± 1.5	6.7 ± 1.4	n/a	n/a	n/a
*T*_lag_ (h)	2.4 ± 0.2	2.0 ± 1.1	5.2 ± 0.9	n/a	n/a	n/a
ΔThT (a.u.)	2067	1286	958	101	0	0

a Aggregation halftime (*t*_1/2_) and lag time (*T*_lag_) were obtained from fitting the ThT curves to eq 1. The increase in ThT fluorescence (ΔThT) is presented in arbitrary fluorescence units (a.u.).

### 2.2 TEM Imaging of Aggregated Aβ_40_ Peptides

**Figure 2:**
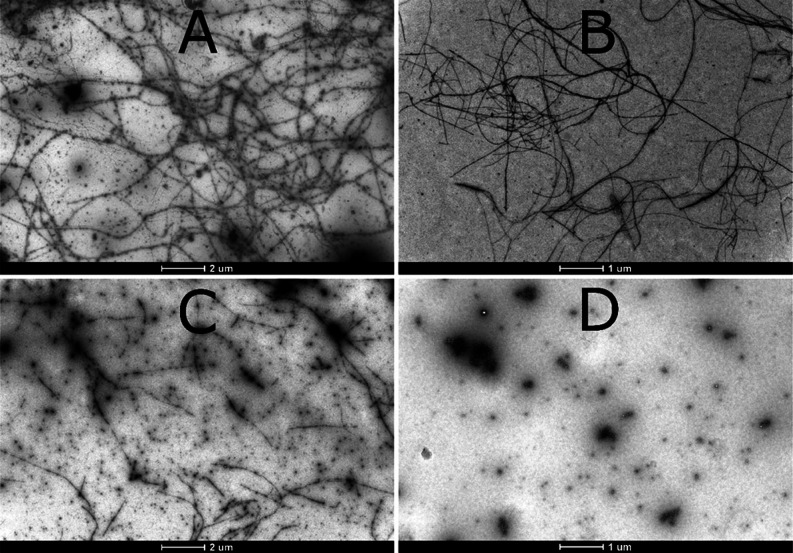
TEM images for aggregates of 20 μM Aβ_40_ in 20 mM MES buffer, pH 7.3, incubated for 20 h on a thermo shaker at 37 °C and 300 rpm, together with different concentrations of uranyl acetate: (A) 0; (B) 0.2; (C) 2; and (D) 20 μM. White scale bars are either 2 (A,C) or 1 μm (B,D).

Negative staining transmission electron microscopy (TEM) images were recorded for 20 μM Aβ_40_ peptide, incubated for 20 h with different concentrations of uranyl acetate, i.e., 0, 0.2, 2, and 20 μM (Figure 2). The uranyl additions correspond to uranyl/Aβ_40_ ratios of 1:100, 1:10, and 1:1. The control sample without UO_2_^2+^ ions displays numerous amyloid fibrils that are several microns long, together with occasional larger clumps and smaller aggregates, which may be proto-fibrils (Figure 2A). This is in line with previous in vitro studies of Aβ_40_ aggregates.^8,25,116^ The Aβ_40_ samples incubated together with 0.2 μM of uranyl acetate display amyloid fibrils of similar size and shape (Figure 2B). In the presence of 2 μM uranyl acetate, there are fewer fibrils and they are shorter, i.e., only a few microns long (Figure 2C). At the highest concentration of uranyl acetate, i.e., 20 μM, no fibrils have formed at all (Figure 2D). Instead, the Aβ_40_ peptides have aggregated into large amorphous clumps, which coexist with smaller particles (Figure 2D). These results show that UO_2_^2+^ ions inhibit the formation of Aβ amyloid fibrils in a concentration-dependent manner, with complete inhibition at stoichiometric uranyl/Aβ_40_ ratios.

### 2.3 NMR Spectroscopy on Uranyl Binding to Aβ_40_ Monomers

**Figure 3:**
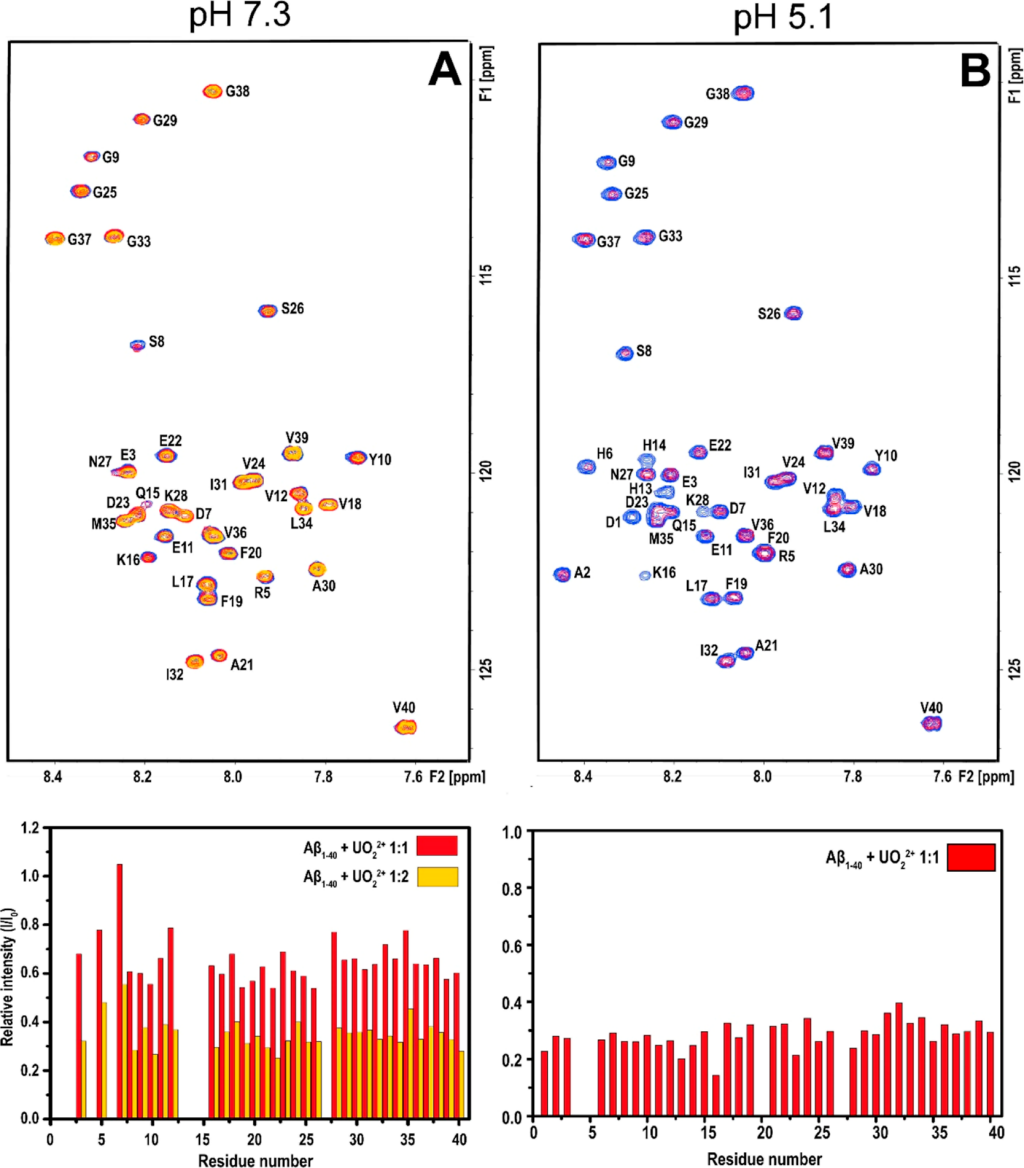
2D NMR ^1^H, ^15^N-HSQC-spectra at 5 °C showing titrations of uranyl acetate to 92 μM monomeric ^15^N-labeled Aβ_40_ peptides in 20 mM MES buffer at either pH 7.3 (A) or pH 5.1 (B). Blue cross-peaks: 84 μM Aβ_40_ only. Red cross-peaks: 1:2 uranyl/Aβ_40_. Yellow cross-peaks (pH 7.3 only): 1:1 uranyl/Aβ_40_. The peak intensities in the bar charts show ratios between the cross-peak intensities with added uranyl ions relative to the intensities before the addition of uranyl ions, i.e., *I*/*I*_0_.

High-resolution nuclear magnetic resonance (NMR) experiments were conducted to investigate if there were residue-specific molecular interactions between uranyl ions and monomeric Aβ_40_ peptides. Figure 3 shows 2D ^1^H, ^15^N-HSQC spectra for the amide cross-peak region for 92 μM ^15^N-labeled Aβ_40_ peptides, at either pH 7.3 or pH 5.1, recorded before and after the addition of uranyl acetate.

At pH 7.3, addition of first 46 μM and then 92 μM uranyl ions (1:2 and 1:1 uranyl/Aβ_40_ ratio, respectively) induces a concentration-dependent loss of amide cross-peak intensity (Figure 3A). The intensity loss is uniformly distributed across the peptide sequence, which shows that the uranyl ions do not bind to specific residues of the Aβ_40_ monomer. Instead, the observed binding is likely driven by general electrostatic interactions between the cationic uranyl ions and the anionic Aβ peptides. The loss of cross-peak intensity is probably caused by several factors, such as paramagnetic quenching effects^99,100^ and intermediate (on the NMR time-scale) chemical exchange between free Aβ_40_ peptides and the Aβ_40_ uranyl complex, similar to the effects observed when Cu(II), Ni(II), and Zn(II) ions bind to Aβ peptides.^117−120^ In addition, the uranyl ions likely promote the formation of Aβ aggregates that either precipitate out of the solution or are too large and heterogeneous to produce distinct NMR signals.^119,120^ As no NMR signals are observed for the Aβ_40_ aggregates, nothing can be concluded about the binding configurations for uranyl ions in complex with such aggregates.

At pH 5.1, addition of uranyl ions again induces a uniform loss of amide cross-peak intensity (Figure 3B). Unexpectedly, the effect is stronger at pH 5.1 than at pH 7.3: addition of 46 μM uranyl acetate (1:2 uranyl/Aβ_40_ ratio) at pH 5.1 decreases the cross-peak intensity approximately as much as the addition of 92 μM uranyl acetate (1:1 ratio) at pH 7.3 (Figure 3). This suggests stronger binding of the uranyl ions at an acidic pH. When comparing the NMR spectra without added uranyl ions at neutral (Figure 3A) and acidic (Figure 3B) pH, NMR cross-peaks for a few additional residues (e.g., D1, A2, H6, H13, and H14) became visible at acidic pH (Figure 3B), probably due to slower proton exchange at this pH.^119^

### 2.4 Fluorescence Measurements of Uranyl Aβ Binding Affinity

**Figure 4:**
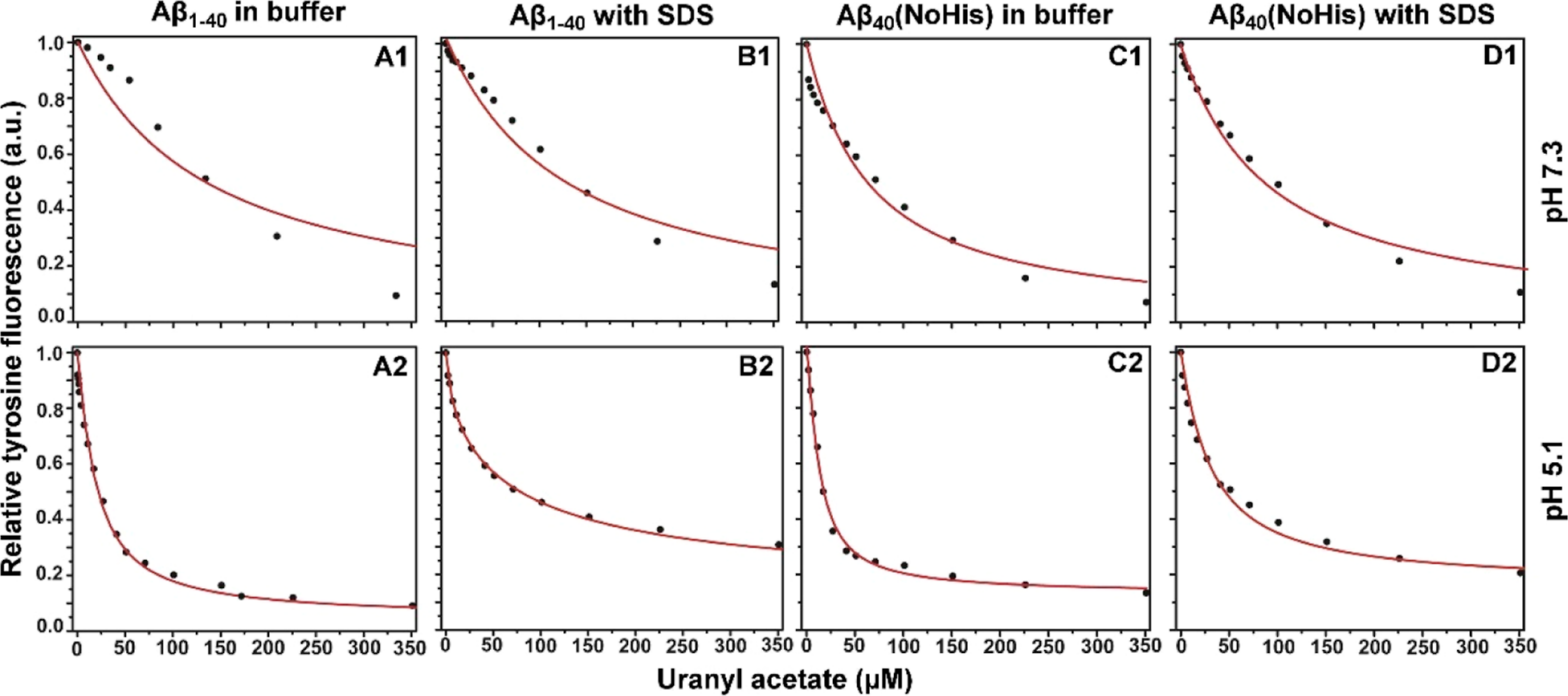
Fluorescence intensity curves for Aβ residue Tyr10, showing titrations of 20 μM Aβ_40_ peptide (A,B) or 20 μM Aβ_40_ (NoHis) mutant (C,D) with uranyl acetate in 20 mM MES buffer, either at pH 7.3 (1st row) or at pH 5.1 (2nd row). For (B,D), micelles of 50 mM SDS were added to the samples.

Uranyl ions were found to quench the intrinsic fluorescence of the Tyr10 residue in the Aβ peptide, similar to, e.g., Cu(II) ions.^121−123^ Measurements of the Tyr10 fluorescence during titrations with uranyl acetate were therefore used to quantify binding affinities for Aβ·UO_2_^2+^ complexes under different conditions. The resulting titration/binding curves are shown in Figure 4. Fitting eq 2 to these curves produced the apparent dissociation constants (*K*_D_^App^) shown in Table 2.

**Table 2:** Apparent Dissociation Constants (*K*_D_^App^) in μM for Uranyl·Aβ Complexes, Obtained by Fitting Fluorescence Titration Curves to eq 2 (Figure 4; Three Replicates per Condition)

	Aβ_40_				Aβ_40_ (NoHis)			
	1	2	3	average	1	2	3	average
pH 7.3	130	141	148	140 ± 8	62	86	79	76 ± 11 μM
pH 7.3 + 50 mM SDS	206	252	251	236 ± 22	119	124	116	120 ± 4 μM
pH 5.1	14.0	13.7	21.3	16.3 ± 4	2.3	2.8	4.0	3.0 ± 1 μM
pH 5.1 + 50 mM SDS	21.8	21.7	27.4	23.6 ± 3	22.3	30.7	27.9	27.0 ± 4 μM

For Aβ_40_ at pH 7.3, the titration data clearly deviate from the binding model, both for the measurements in buffer only (Figure 4A1) and in the presence of sodium dodecyl sulfate (SDS) micelles (Figure 4B1). Because of these deviations, the derived dissociation constants should not be trusted. As eq 2 is based on a model that assumes a single binding site, a possible explanation for this deviation is the binding of uranyl ions to multiple locations on the Aβ_40_ peptide under these conditions. At pH 5.1, the binding curves follow the model very well (Figure 4A2,B2), yielding reliable *K*_D_^App^ values of 16.3 μM for Aβ_40_ in buffer and 23.6 μM for Aβ_40_ bound to SDS micelles (Table 2). The main difference from the measurements at neutral pH is that the three His residues in the Aβ peptide become protonated at lower pH as their p*K*_a_ values are around 6.8.^124^ Because protonated His residues are unlikely to interact with positive ions such as UO_2_^2+^, it is possible that binding interactions between uranyl ions and uncharged His residues are responsible for the deviations from the single-site binding scheme observed at pH 7.3 (Figure 4A1,B1).

The binding curves for the Aβ_40_ (NoHis) mutant at pH 7.3 follow the model rather well (Figure 4C1,D1), producing *K*_D_^App^ values of 76 μM in buffer and 120 μM in the presence of SDS micelles (Table 2). At pH 5.1, the overlap with the fitted curve is even better (Figure 4C2,D2), and here, the *K*_D_^App^ values are 3.0 μM in buffer and 27 μM in the presence of SDS micelles. With these results, some comparisons can be made.

For both peptide versions, the uranyl ions display stronger binding at low pH. Even though reliable binding constants could not be obtained for Aβ_40_ at pH 7.3, it is clear from the measurement data that binding is overall stronger at pH 5.1 (Figure 4A,B). Addition of SDS micelles generally makes the binding weaker (Table 2). This effect is likely related to the SDS molecules being negatively charged, thereby competing for binding to the cationic uranyl ions. The strongest uranyl binding is observed for the Aβ_40_ (NoHis) mutant at pH 5.1 (3.0 μM), while the weakest binding is observed for Aβ_40_ at pH 7.3. It therefore appears that neutral (i.e., non-protonated) His residues might interfere with uranyl binding under the experimental conditions used.

### 2.5 CD Spectroscopy on the Aβ Secondary Structure

**Figure 5:**
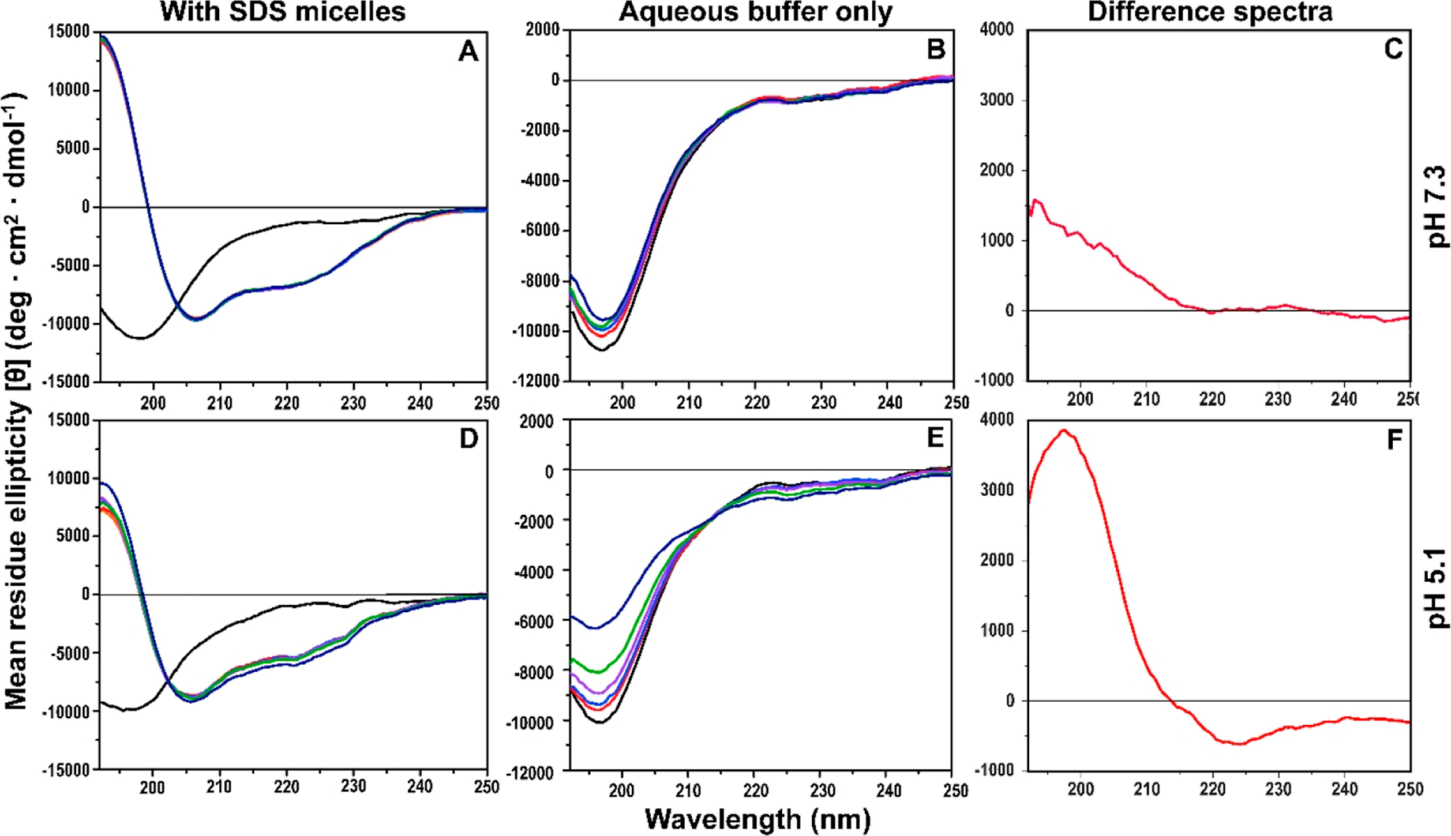
CD spectroscopic titrations of 10 μM Aβ_40_ with uranyl acetate in 20 mM sodium phosphate buffer at 20 °C, carried out in the presence of 50 mM SDS at pH 7.3 (A) or pH 5.1 (D), or in aqueous buffer only at pH 7.3 (B) or pH 5.1 (E). First, spectra were recorded for Aβ_40_ in buffer for all samples (black lines). Then, 50 mM SDS detergent was added to the samples in (A,D) (orange lines). To all samples, uranyl acetate was added in steps of 2, 6, 16, 56, and 256 μM (ruby red, ultramarine blue, plum purple, lime green, and navy blue lines, respectively). For the titrations in aqueous buffer, shown in (B,E), the difference spectra shown in (C,F) were created by subtracting the measurements with 256 μM added uranyl from the measurements without uranyl, at pH 7.3 (C) and pH 5.1 (F).

Circular dichroism (CD) spectroscopy was used to investigate the possible effects of uranyl ions on the secondary structure of Aβ_40_ peptides, both in aqueous buffer and in the presence of SDS micelles that mimic a membrane environment. The CD spectra for Aβ_40_ monomers in aqueous buffer display spectra with minima around 196–198 nm (Figure 5B,E), which is typical for a random coil conformation. At pH 5.1, addition of uranyl ions to Aβ_40_ induces a two-step structural transition as the CD signal changes in a concentration-dependent manner around an isodichroic point at approximately 214 nm (Figure 5E). The difference spectrum (Figure 5F) shows that the structural transition is a conversion from random coil to β-sheet. At pH 7.3, the structural effect induced by the uranyl ions is weaker, and it is unclear if an isodichroic point is present (Figure 5B). The difference spectrum is not conclusive but might represent β-sheet conformation (Figure 5C).

In the presence of SDS micelles, the CD signals for Aβ_40_ peptides display minima around 208 and 222 nm (Figure 5A,D), which is characteristic for the α-helical secondary structure. This is in line with previous reports that the central and C-terminal Aβ regions adopt α-helical conformations in membrane-like environments.^19,122,125,126^ At pH 7.3, addition of uranyl ions induces no changes at all in the Aβ_40_ CD spectrum with SDS (Figure 5A). At pH 5.1, however, the uranyl ions induce systematic changes in the CD spectra around an isodichroic point at approximately 203 nm (Figure 5D). This is the same wavelength as where the CD spectrum for Aβ_40_ peptides in aqueous solution (i.e., random coil structure) crosses the CD spectrum for Aβ_40_ peptides in SDS micelles (i.e., α-helical structure), when no uranyl ions are present (Figure 5D). Taken together, these observations indicate that uranyl ions at pH 5.1 induce a structural conversion from α-helix to a random coil in Aβ_40_ peptides in SDS micelles. Also, the changes in CD intensity at other wavelengths are consistent with the formation of a random coil structure, even though the overall changes are small and difficult to interpret conclusively (Figure 5D). The overall larger structural effects induced by uranyl ions at acidic pH, compared to neutral pH, both in aqueous solution (Figure 5B,E) and in SDS micelles (Figure 5A,D), support the NMR (Figure 3) and fluorescence (Figure 4) spectroscopy results that the uranyl ions bind stronger to Aβ_40_ peptides at acidic pH.

### 2.6 Dityrosine Cross-Link Formation

**Figure 6:**
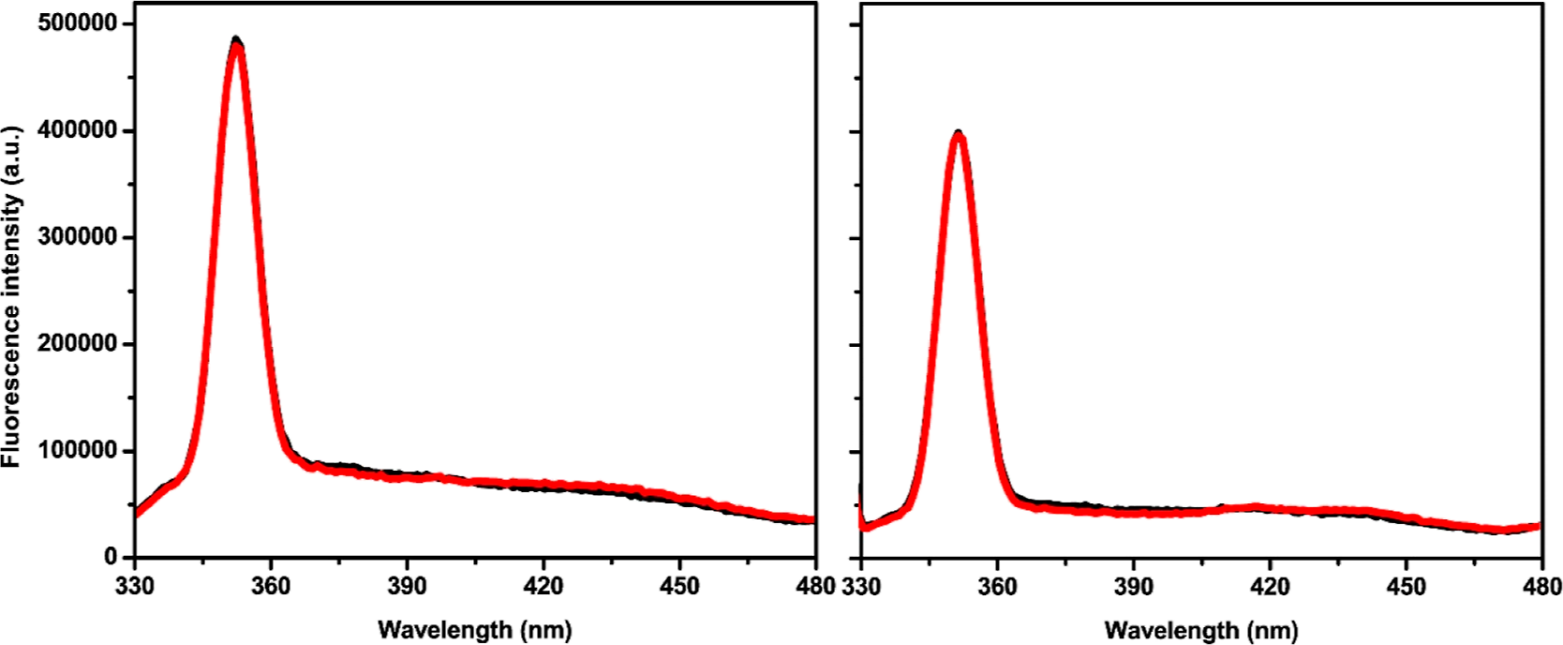
Fluorescence emission spectra of 10 μM Aβ_40_ peptides in 20 mM MES buffer, pH 7.3, incubated with 100 μM EDTA (A) or with 100 μM uranyl acetate (B). Black line—0 h and red line—24 h.

Fluorescence measurements were carried out to investigate if uranyl ions could induce the formation of covalent dityrosine cross-links in Aβ peptides via the generation of ROS, similar to what has been observed for redox-active Cu(II) ions^35,127−129^ and Ni(II) ions.^118^ Two samples of Aβ_40_ peptides were studied. The control sample contained 100 μM EDTA to remove possible contaminating metal ions that could promote dityrosine formation. The other sample contained 100 μM uranyl acetate. For both samples, the fluorescence spectra are virtually identical before and after 24 h of incubation (Figure 6). Specifically, no peak around 410 nm, indicative of dityrosine,^129,130^ has emerged. The signal around 350 nm is a water Raman peak, which is useful for reference purposes as it does not change with the sample composition. These results clearly show that uranyl ions do not induce the formation of dityrosine cross-links under the experimental conditions employed here.

### 2.7 BN-PAGE Analysis of Aβ_42_ Oligomer Formation and Stability

Blue native polyacrylamide gel electrophoresis (BN-PAGE) analysis and Fourier-transform infrared (FTIR) analysis (below) were used to investigate possible effects of uranyl ions on Aβ oligomer formation. Because Aβ_40_ peptides do not form stable oligomers, Aβ_42_ peptides together with SDS detergent were used to create oligomers that remained stable for over a week. The Aβ_42_ oligomers were created with two different concentrations of stabilizing SDS molecules, 80–100 μM of Aβ_42_ peptides and 0–1000 μM of uranyl acetate, as described in Section 5.2.

**Figure 7:**
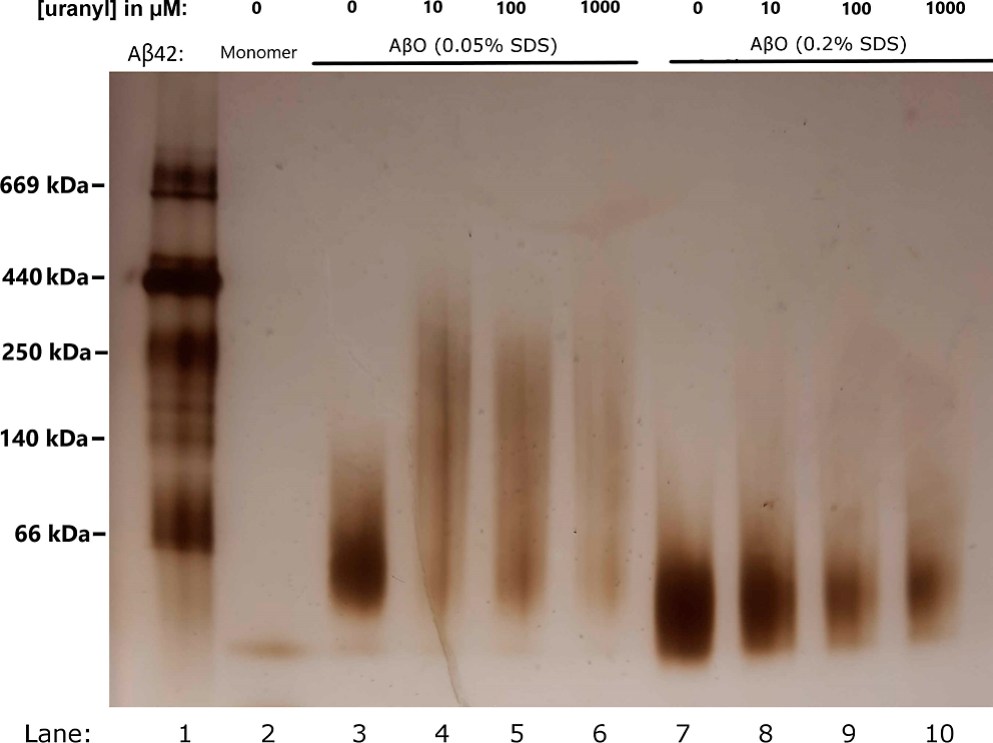
BN-PAGE gel showing the effects of different concentrations of uranyl acetate on the formation of SDS-stabilized Aβ_42_ oligomers (formed with 80–100 μM peptide). Lane 1: reference ladder. Lane 2: Aβ_42_ monomers. Lanes 3–6: AβO_0.05%SDS_ oligomers (likely dodecamers) prepared with 0, 10, 100, and 1000 μM uranyl ions, respectively. Lanes 7–10: AβO_0.2%SDS_ oligomers (likely tetramers) prepared with 0, 10, 100, and 1000 μM uranyl ions, respectively.

The BN-PAGE analysis shows that in 0.05% SDS (1.7 mM) without uranyl acetate, larger oligomers with a molecular weight (MW) around 55–60 kD are formed (Figure 7, lane 3). These larger oligomers, abbreviated AβO_0.05%SDS_, most likely contain twelve Aβ_42_ peptides and display a globular morphology, which is why they are sometimes called globulomers.^131^ In the presence of different concentrations of uranyl acetate, no well-defined uniform oligomers were observed. Instead, the bands on the BN-PAGE gel appear smeared, indicating a heterogeneous size distribution of the formed oligomers (Figure 7, lanes 4–6). This disruptive effect is already very strong at the lowest concentration (10 μM) of added uranyl ions.

In 0.2% SDS (6.9 mM), small Aβ_42_ oligomers with a MW around 16–20 kDa are formed (Figure 7, lane 7). These oligomers, abbreviated AβO_0.2%SDS_, likely contain a large fraction of tetramers.^131,132^ Preparation of these oligomers in the presence of increasing uranyl concentrations (10, 100, and 1000 μM) yields oligomer bands that are weaker at the intermediate uranyl concentrations and weakest at the highest concentration, indicating gradually lower amounts of stable AβO_0.2%SDS_ oligomers (Figure 7, lanes 8–10). Thus, for the formation of the smaller AβO_0.2%SDS_ oligomers at the higher SDS concentration, the effect of uranyl ions is less disruptive and clearly concentration-dependent compared to the effect on the larger AβO_0.05%SDS_ oligomers (Figure 7).

### 2.8 FTIR Spectroscopy Reflecting the Aβ_42_ Oligomer Structure

The secondary structures of Aβ_42_ oligomers formed with different SDS and uranyl concentrations were studied with FTIR spectroscopy, where the amide I region (1700–1600 cm^–1^) is very sensitive to changes in the protein backbone conformation, including differences in β-sheet structures.^133−135^ When prepared in the absence of uranyl acetate, both types of Aβ_42_ oligomers (i.e., AβO_0.05%SDS_ and AβO_0.2%SDS_) produce two major bands in the amide I region: a high-intensity band near 1630 cm^–1^ and a smaller one near 1686 cm^–1^.^132^ This split pattern of IR bands in the amide I region is typical for anti-parallel β-sheet structures.^136−138^

**Figure 8:**
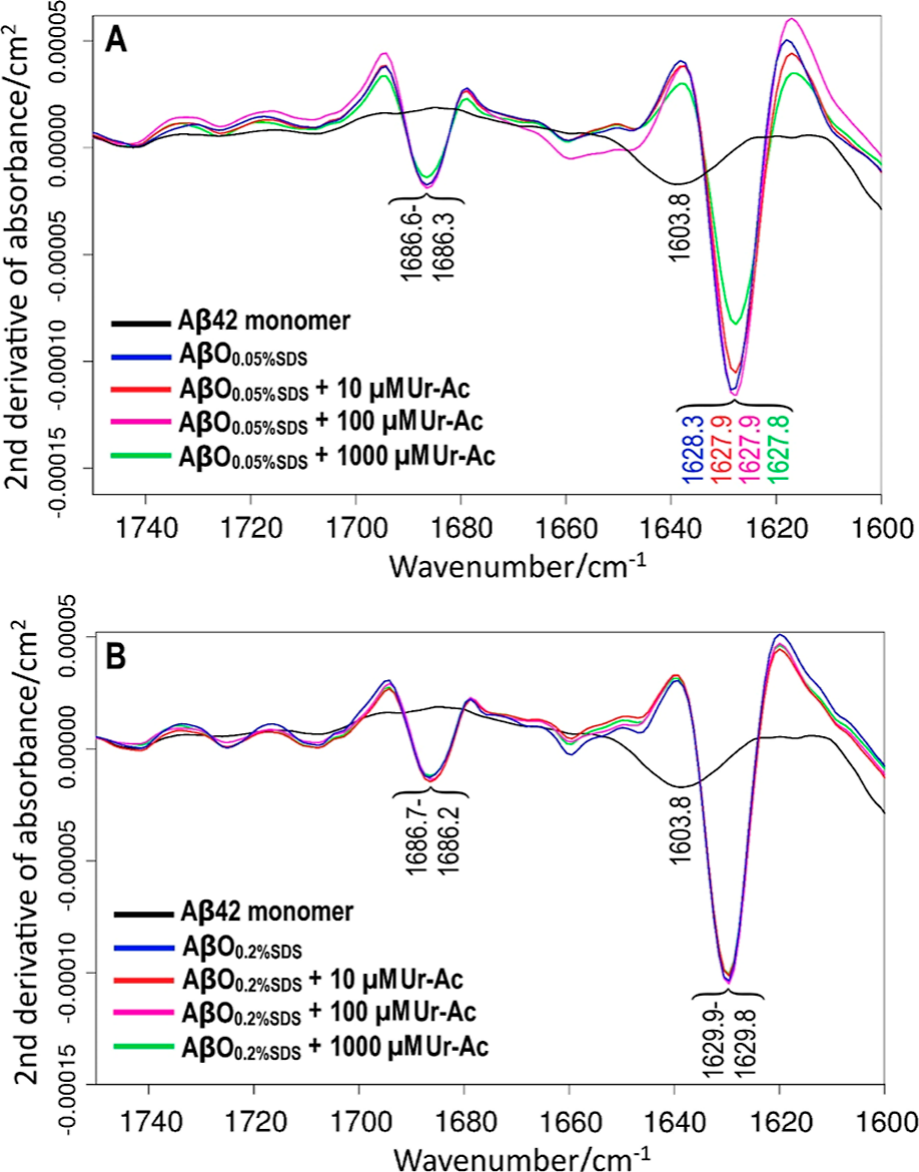
Second derivatives of infrared absorbance spectra for 80–100 μM SDS-stabilized Aβ_42_ oligomers formed in the absence (blue) and presence of 0.01 mM (red), 0.1 mM (purple), and 1 mM (green) uranyl acetate. The results are shown for larger Aβ_42_ oligomers formed with 0.05% SDS (A) and for smaller Aβ_42_ globulomers formed with 0.2% SDS (B).

For AβO_0.05%SDS_ oligomers in PBS buffer, the β-sheet main band shows a band shift due to uranyl acetate: the main band is observed at 1628.3 cm^–1^ without uranyl acetate, but shifts down to 1627.8 cm^–1^ in the presence of 1 mM (1000 μM) uranyl acetate (Figure 8A). In contrast to the AβO_0.05%SDS_ oligomers, the spectrum of the AβO_0.2%SDS_ oligomers (resolved at 1629.9 cm^–1^, Figure 8B) is not significantly affected by the uranyl acetate concentration (1629.8 cm^–1^ at 0.01, 0.1, and 1 mM uranyl acetate) (Figure 8B). This result also indicates that band positions can be determined with an accuracy of 0.1 cm^–1^, which is supported by our study of Li(I) ion effects,^139^ where at both SDS concentrations, the standard deviation of the band positions for four Li(I) ion concentrations (0, 0.1, 1, and 10 mM) was 0.12 cm^–1^ and the band positions at zero and 10 mM Li(I) differed by less than 0.1 cm^–1^.

These observations agree with the BN-PAGE results, which showed clear small oligomer bands for all uranyl concentrations at the higher SDS concentration, but extended smears instead in the presence of uranyl acetate for the lower SDS concentration. The results indicate again that the Aβ_42_ globulomers produced at the lower SDS concentration are more sensitive to uranyl-induced effects.

## 3 Discussion

Uranium is a well-known neurotoxicant,^39^ but the underlying molecular mechanisms and a possible role of U in neurodegenerative diseases remain unclear.^49,91^ For AD, several studies have investigated how Aβ peptides interact with various metal ions^30,47,118,139−142^ and with small cationic molecules.^28,143^ We here interpret the current results on Aβ–uranyl interactions in the light of this earlier work.

### 3.1 Effects of Uranyl Ions on Aβ_40_ Aggregation

The ThT fluorescence curves (Figure 1) and TEM images (Figure 2) show that uranyl ions have a concentration-dependent inhibitory effect on the fibrillization of Aβ_40_ peptides at physiological pH. Instead of forming proper amyloid fibrils, the Aβ_40_ peptides form non-fibrillar amorphous aggregates (clumps) already at a 1:1 uranyl/Aβ_40_ ratio (Figure 2). This effect is seen both in the TEM images and the ThT fluorescence experiments, even though the sample conditions are somewhat different (i.e., the TEM samples did not contain ThT dye and were incubated in Eppendorf tubes on a thermo shaker). Thus, the uranyl ions do not prevent the Aβ_40_ peptides from aggregating but rather direct the aggregation process toward non-fibrillar end-products. Similar effects on the Aβ aggregation pathways have previously been observed for other small cationic molecules and heavy metal ions such as Hg(II), Ni(II), Pb(II), and Pb(IV).^47,118,143^ As small oligomeric aggregates of Aβ peptides are considered to be the main toxic species in AD pathology,^2,11^ the finding that uranyl ions can modulate Aβ aggregation might be relevant for understanding AD progression and pathogenesis.

### 3.2 Binding of Uranyl Ions to Aβ_40_ Peptides

The NMR results show that uranyl ions have no obvious residue-specific binding to Aβ_40_ peptides (Figure 3). Because of the strong covalent O=U=O bonds in the uranyl ion, it is mainly the central U(VI) atom that interacts with other molecules.^144^ Thus, the uranyl–Aβ binding is probably mediated via non-specific electrostatic interactions between the positive U(VI) atom and negative Aβ residues, such as Asp1, Asp7, Asp23, Glu3, Glu11, and Glu22. This is similar to Aβ binding to Mn(II) and Pb(II) ions,^3,140^ but different from numerous small cationic species that display residue-specific interactions with Aβ peptides, such as polyamines^28^ and Cu(II), Ni(II), Pb(IV), and Zn(II) ions.^3,117−119^

The results of the NMR measurements indicate that the Aβ binding of uranyl ions is stronger at acidic pH (Figure 3), which is confirmed by the fluorescence measurements of uranyl–Aβ binding affinity (Figure 4) and also by the CD spectroscopy results (Figure 5). At pH 5.1, the apparent binding affinity of uranyl ions is 16.3 ± 4 μM to Aβ_40_ peptides and 3.0 ± 1 μM to the Aβ_40_ (NoHis) mutant (Table 2). At neutral pH, the binding is about a magnitude weaker (Table 2) and also less well-defined (Figure 4). This is counter-intuitive. The main chemical difference at lower pH is that the Aβ His residues become protonated as they have p*K*_a_ values around 6.8 in short peptides.^124^ It stands to reason that Aβ peptides with positively charged His residues should be less prone to interact with positive metal ions, as has been shown for, e.g., Cu(II) and Zn(II) ions.^119^ On the other hand, the uranyl ion is a weak acid that undergoes hydrolysis, and the first p*K*_a_ is around 4–5.^145,146^ Thus, at pH 5.1, the uranyl ions may be mixed with monomeric and dimeric hydroxide species. At pH 7.3, additional species might be present, such as the single-charged trimer [(UO_2_)_3_(OH)_5_]^+^. It is possible that such heterogeneity, which is likely more pronounced at neutral pH, could help explain the somewhat unexpected uranyl titration results.

The Tyr10 fluorescence measurements (Figure 4) show that the uranyl Aβ binding affinity increases at lower pH values and also when the three His residues are replaced by Ala residues (Table 2). In fact, the weakest binding is observed for Aβ_40_ at pH 7.3, and the strongest binding is observed for the Aβ_40_ (NoHis) mutant at pH 5.1 (Table 2). This trend is observed for Aβ samples both in aqueous buffer and in SDS micelles. It strongly suggests that the presence of uncharged His sidechains interferes with uranyl binding to Aβ peptides (Table 2). This tentative conclusion is supported by the shape of the fluorescence data curves, which are more uniform and better fit the single-binding-site model (eq 2), when the uncharged His residues H6, H13, and H14 are replaced with Ala residues in the Aβ_40_ (NoHis) mutant (Figure 4). In addition, at acidic pH, the uranyl binding is stronger both to the wild-type (wt) Aβ_40_ peptide and to the Aβ_40_ (NoHis) mutant. In the latter case, the effect is clearly not caused by His protonation as these residues are missing in the mutant peptide. Thus, the His residues are not the only factor responsible for the unusual properties of uranyl-Aβ binding. As discussed above, formation of uranyl hydroxide species is likely another confounding factor. The molecular details of uranyl-Aβ binding should therefore be further explored in future studies.

Because toxic Aβ aggregates appear to form in membrane environments,^147^ and as membrane disruption might be one of the toxic mechanisms of Aβ aggregates,^148^ it is important to clarify how Aβ interacts with uranyl ions also in a membrane environment. Here, we used SDS micelles as a simple membrane model. Even though such micelles are different from the phospholipid bilayers present in cell membranes, they do share some properties with real membranes, while being much more suitable for various spectroscopy measurements, including NMR.^125,126^ Our fluorescence spectroscopy measurements showed that the uranyl binding affinities decrease slightly when SDS micelles are present in the sample for all Aβ variants and conditions (Figure 4 and Table 2). This effect is probably caused by the anionic SDS micelles competing for binding to the cationic uranyl ions. Similar results have earlier been obtained for Aβ binding to other metal ions, such as Cu(II), Hg(II), and Ni(II), in the presence of SDS micelles.^47,118,122^ Aβ peptides are known to insert only their central and C-terminal regions into an SDS micelle, while the negatively charged N-terminal region is positioned outside the micelle where it is free to interact with, e.g., cations.^118,122^ Thus, it appears that Aβ peptides can (sometimes) bind uranyl ions mainly via their N-terminal part in a membrane environment. We may speculate that when multiple Aβ peptides are present in the membrane, a single uranyl ion might bind to the N-termini from two or more Aβ peptides, thereby bringing the peptides together and promoting aggregation.

### 3.3 Effects of Uranyl Ions on the Aβ_40_ Peptide Structure

In an aqueous buffer at pH 5.1, the CD spectroscopy measurements show that uranyl ions induce a two-state structural transition in the Aβ_40_ peptides, from random coil to β-sheet structure (Figure 5E,F). At pH 7.3, a weaker structural transition is observed, which might be a similar conversion into a β-sheet structure (Figure 5B,C). Earlier studies have shown that such structural changes can be induced also by metal ions such as Cu(II), Ni(II), and Zn(II).^118,119^ Because Aβ aggregates typically consist of peptides in β-sheet conformation,^18^ this type of β-sheet structure formation likely promotes Aβ aggregation.

SDS micelles constitute a simple membrane model,^125,126^ and the central and C-terminal regions of Aβ peptides are known to insert themselves into such micelles and adopt α-helical conformations.^19,126^ In the presence of SDS micelles, uranyl ions have no effect on the Aβ_40_ peptide conformation at pH 7.3 (Figure 5A). However, a weak structural transition is observed at pH 5.1, possibly involving the formation of random coils (Figure 5D). Previous studies have found that metal ions that bind to Aβ peptides mainly via the His residues, such as Cu(II), Ni(II), and Zn(II) ions, can induce altered coil–coil interactions in Aβ peptides positioned in SDS micelles.^118,122^ No such alterations in the α-helical structure were observed upon the addition of uranyl ions (Figure 5A,D).

No dityrosine cross-links were observed after Aβ_40_ peptides had been incubated together with uranyl acetate (Figure 4). Earlier studies have shown that redox-active metal ions such as Cu(II) and Ni(II) can induce the formation of dityrosine cross-links in amyloid peptides via redox-cycling of, e.g., the Cu(I)/Cu(II) redox pair, which generates harmful oxygen radicals via Fenton-like chemistry.^35,37,38,118,129^ For short peptides with only one Tyr residue in the amino acid sequence, such as Aβ and amylin, dityrosine formation must involve two peptides, which then become linked to form a dimer.^129^ As Cu(II) and Ni(II) bind Aβ peptides mainly via the His6, His13, and His14 residues, it is likely that these metal ions can promote Aβ aggregation by coordinating multiple His residues from more than one Aβ peptide.^141,149^ Cu(II) and Ni(II) ions may therefore promote dityrosine formation not only by creating oxygen radicals but also by connecting two Aβ molecules and positioning their Tyr10 residues close to each other. It is known that the U(VI) ion in uranyl can be reduced to lower valency states such as U(IV) under appropriate reducing conditions.^144,150^ Thus, as uranyl acetate has been found to induce oxidative stress in isolated cells,^97^ the uranyl ions are likely redox-active under physiological conditions. We therefore speculate that the reason why uranyl ions do not promote dityrosine formation in Aβ_40_ peptides (Figure 4) is the weak and non-specific binding under the experimental conditions used (Figures 7, 8, and Table 2).

### 3.4 Effects of Uranyl Ions on Aβ_42_ Oligomers

Although most of the measurements carried out in this study were performed on Aβ_40_ monomers, it is also of interest to investigate the possible effects of uranyl ions on Aβ oligomers. Because Aβ_40_ peptides do not form stable oligomers, BN-PAGE and FTIR studies were carried out on Aβ_42_ oligomers stabilized by SDS detergent. The BN-PAGE experiments clearly show that uranyl ions interfere with the formation of homogeneous Aβ_42_ oligomers (Figure 7). This is further supported by the FTIR measurements, where the position of the β-sheet main band is downshifted when increasing concentrations of uranyl ions are present during oligomer formation (Figure 8). The uranyl effect is more pronounced on the larger and mainly dodecameric Aβ_42_ oligomers, which are formed in the presence of 0.05% SDS. The spectral changes observed with uranyl ions are consistent with the previously observed effects of Ni(II) ions.^118^ However, only 10 μM of divalent uranyl ions (Figure 7, Lane 4) but 500 mM of divalent Ni(II) ions^118^ are required for full disruption of homogeneous AβO_0.05%SDS_ oligomers, even though Ni(II) ions display a stronger binding affinity for Aβ peptides than uranyl ions at neutral pH (Figures 7, 8 and Table 2). In general, the effects of uranyl ions on Aβ oligomerization qualitatively resemble those of other transition metal ions, including Ni,^118^ more than those of monovalent alkali ions, such as Li.^139^ Such conclusions are consistent with the theoretical findings regarding the relative propensities of different metal ions for interactions with polypeptides.^151^

### 3.5 Medical Implications

Our current results show that uranyl ions induce structural changes in Aβ monomers and Aβ oligomers and inhibit Aβ fibrillization and homogeneous oligomer formation already at sub-stoichiometric concentrations. It is unclear how these uranyl interactions may influence (or not) the toxicity of Aβ oligomers or the Aβ-induced pathology in AD patients. Furthermore, no link has been established between uranium/uranyl exposure and AD incidence. On the other hand, very few people have investigated such possible links. The current results show that uranyl ions affect Aβ peptide aggregation in similar ways as Pb and Hg ions, and Pb and Hg exposure might be linked to the development of AD and other neurodegenerative diseases.^3,47^ Given these similar molecular interactions, it could be worthwhile to conduct cell studies, animal studies, and epidemiological studies on AD patients to find out if exposure to uranium/uranyl might induce AD. However, even if such an effect does exist, U is a well-known toxic metal, and it is possible that people exposed to U will suffer more immediate harmful health effects that will mask a slowly progressing dementia. The molecular mechanisms for the chemical toxicity of U are not fully understood. Our current results show that even low concentrations of uranyl can induce unstructured protein aggregation in Aβ peptides and, most likely, also in other peptides and proteins. Thus, a general toxic mechanism of uranyl ions could be to modulate protein folding, misfolding, and aggregation.

## 4 Conclusions

Uranyl ions, UO_2_^2+^, bind to Aβ_40_ peptides via non-specific electrostatic interactions, with an apparent binding affinity of 16.3 ± 4 μM at pH 5.1. Uranyl binding is weaker and less uniform at neutral pH, possibly because of interference from His sidechains and from other uranyl species such as hydroxides. The uranyl ions inhibit Aβ fibrillization and oligomer formation in a concentration-dependent manner, with clear effects already at sub-stoichiometric concentrations, and also induce structural changes in Aβ monomers and Aβ oligomers. A general toxic mechanism of uranyl ions could be to modulate protein folding, misfolding, and aggregation.

## 5 Materials and Methods

### 5.1 Materials

SDS detergent, 2-(*N*-morpholino)ethanesulfonic acid (MES) hydrate, sodium phosphate, depleted uranyl acetate, and dimethyl sulfoxide (DMSO) were all purchased from Sigma-Aldrich (USA).

Synthetic lyophilized wt Aβ (1–42) peptides, abbreviated as Aβ_42_, with the primary sequence DAEFR_5_HDSGY_10_EVHHQ_15_KLVFF_20_AEDVG_25_SNKGA_30_IIGLM_35_VGGVV_40_IA, were purchased from JPT Peptide Technologies (Germany). Two recombinant Aβ variants were purchased as lyophilized powder from AlexoTech AB (Umeå, Sweden), namely, the Aβ_40_ peptide and the Aβ_40_ (H6A, H13A, and H14A) triple-mutant, which in the following is referred to as the Aβ_40_ (NoHis) mutant. The Aβ_40_ peptide was also purchased uniformly single-labeled with ^15^N isotopes. All Aβ_40_ peptide variants were stored at −80 °C until use. Before measurements, they were dissolved in 10 mM NaOH and then sonicated in an ice bath for 5 min to avoid pre-formed aggregates. The samples were then diluted in either sodium phosphate buffer at pH 7.3 or in MES buffer at pH 7.3 or 5.1. The peptide concentrations were initially estimated from the weight of the dry powder and then more accurately determined with a NanoDrop spectrophotometer.

### 5.2 Preparation of Aβ_42_ Oligomers

Treatment of Aβ_42_ peptides with low concentrations (≤7 mM) of SDS, i.e., below the critical micelle concentration for SDS, which is 8.2 mM in water at 25 °C,^152^ leads to the formation of stable and homogeneous Aβ_42_ oligomers of certain sizes and conformations.^131,132,153^ To prepare such oligomers, size exclusion chromatography (SEC) was initially used to purify synthetic Aβ_42_ peptides into monomeric form. First, 1 mg of lyophilized Aβ_42_ powder was dissolved in 250 mL of DMSO. Next, a Sephadex G-250 HiTrap desalting column (GE Healthcare, Uppsala) was equilibrated with a 5 mM NaOH solution (pH = 12.3) and washed with a solution of 10–15 mL of 5 mM NaOD, pD = 12.7.^154^ The peptide solution in DMSO was applied to the column, followed by an injection of 1.25 mL of 5 mM NaOD. The collection of peptide fractions in 5 mM NaOD on ice was started at a 1 mg/ mL flow rate. Ten fractions of 1 mL volumes were collected in 1.5 mL Eppendorf tubes. The absorbance for each fraction at 280 nm was measured with a NanoDrop instrument (Eppendorf, Germany), and peptide concentrations were determined using a molar extinction coefficient of 1280 M^–1^ cm^–1^ for the single Tyr in Aβ_42_.^155^ The peptide fractions were flash-frozen in liquid nitrogen, covered with argon gas on top in 1.5 mL Eppendorf tubes, and stored at −80 °C until used. SDS-stabilized Aβ_42_ oligomers of two well-defined sizes (approximately tetramers and dodecamers) were prepared according to a previously published protocol,^131^ but in D_2_O, at a 4-fold lower peptide concentration and without the original dilution step.^132^ The reaction mixtures [100–120 μM Aβ_42_ in PBS, containing either 0.05% (1.7 mM) SDS or 0.2% (6.9 mM) SDS] were incubated together with 0–1000 μM uranyl acetate at 37 °C for 24 h and then flash-frozen in liquid nitrogen and stored at −20 °C for later analysis.

### 5.3 Thioflavin T Aggregation Kinetics

To monitor the effect of uranyl ions on Aβ_40_ aggregation kinetics, a FLUOstar Omega microplate reader (BMG LABTECH, Germany) was used. Samples containing 20 μM Aβ_40_wt, 20 mM MES buffer pH 7.3, 50 μM thioflavin T, and different concentrations of uranyl acetate (i.e., 0, 0.04, 0.2, 0.4, 2, and 20 μM) were added to a 384-well plate, with 35 μL of sample in each well. Thioflavin T is a benzothiazole dye that increases in fluorescence upon binding to amyloid aggregates^116^ and is therefore used to monitor the formation of amyloid aggregates. The ThT dye was excited at 440 nm, and ThT fluorescence emission at 480 nm was measured every 5 min. Before each measurement, the plate was shaken in orbital mode for 140 s at 200 rpm. The samples were incubated for a total of 15 h, and the assay was repeated three times with four replicates of each condition. To determine kinetic aggregation parameters, the data was fitted to eq 1:
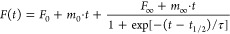
1Here, *F*_0_ and *F*_∞_ are the intercepts of the initial and final fluorescence intensity baselines, *m*_0_ and *m*_∞_ are the slopes of the initial and final baselines, *t*_1/2_ is the time needed to reach halfway through the elongation phase (i.e., aggregation half-time), and τ is the elongation time constant.^116^

### 5.4 Transmission Electron Microscopy Imaging

Negative staining TEM images were recorded for Aβ_40_ peptides that had aggregated under the same conditions as in the ThT fluorescence studies (above). Thus, 20 μM of Aβ_40_ in 20 mM MES buffer, pH 7.3, was incubated for 20 h on a thermo shaker at 37 °C and 300 rpm, together with 0, 0.2, 2, and 20 μM of uranyl acetate. Then, samples of 5 μL were put on copper grids of 200 μm mesh size, which were covered with a Pioloform film upon which a carbon layer had been deposited and then glow-discharged with a Leica EM ACE600 carbon coater (Leica Microsystems, Germany). The Aβ_40_ samples were absorbed onto the grids for 5 min, rinsed with Milli-Q water two times, and then stained for 2 min with a 2% aqueous solution of uranyl acetate. Next, the excess stain was removed with filter paper, and the samples were left to air-dry. A digital Orius SC1000 camera was used to record TEM images in a FEI Tecnai G2 Spirit electron microscope (FEI, The Netherlands) operating at 120 kV accelerating voltage.

### 5.5 Circular Dichroism Spectroscopy Measurements of the Secondary Structure

CD measurements were carried out on a Chirascan CD spectrometer from Applied Photophysics Ltd. (U.K). Samples containing 600 μL of 10 μM Aβ_40_ peptide in 20 mM phosphate buffer, at either pH 7.3 or pH 5.1, were placed in a quartz cuvette with a 2 mm pathlength. CD spectra were recorded at 20 °C between 192 and 250 nm using steps of 0.5 nm and a sampling time of 5 s per data point. Then, small volumes of uranyl acetate were titrated to the samples in steps of 2, 6, 16, 56, and finally 256 μM. The total increase of volume upon the addition of the uranyl acetate was less than 3%. All data was processed with the Chirascan Pro-Data v.4.4.1 software (Applied Photophysics Ltd., U.K.), including smoothing with a ten-point smoothing filter. 50 mM SDS detergent was added to some of the samples as SDS micelles constitute a simple model for bio-membranes.^125,126^ Aβ peptides are known to insert their central and C-terminal segments as α-helices into SDS micelles, while the N-terminal Aβ segment remains unstructured outside the micelle surface.^19,125^ Because the critical micelle concentration for SDS is 8.2 mM in water at 25 °C,^152^ micelles clearly formed under the experimental conditions. With approximately 62–65 SDS molecules per micelle,^156^ 50 mM SDS yields a micelle concentration slightly below 1 mM, i.e., much higher than the concentration of Aβ peptides. This means that each micelle will generally contain no more than one Aβ peptide, which effectively prevents Aβ aggregation and ensures that uranyl interactions are with monomeric Aβ peptides. The high SDS concentration used to obtain this condition, i.e., 50 mM, does not pose a problem in the current experiments. Even though 50 mM SDS would efficiently denature most folded proteins, such denaturing effects are not relevant for small intrinsically disordered peptides such as Aβ (in monomeric form).

### 5.6 Fluorescence Measurements of Dityrosine Formation

Fluorescence emission spectra between 330 and 480 nm (excitation at 315 nm) were recorded at room temperature with a Jobin Yvon Horiba Fluorolog 3 fluorescence spectrometer (Longjumeau, France) for two samples of 10 μM Aβ_40_ peptide in 20 mM MES buffer, pH 7.3. One sample contained 100 μM uranyl acetate to investigate the possible effects of uranyl ions on dityrosine formation. The control sample contained 100 μM of the chelator EDTA to remove any free metal ions. All measurements were conducted in triplicate using a quartz cuvette with a 4 mm path length and containing a 0.7 mL liquid sample. Spectra were recorded after 0 and 24 h of incubation, during which the samples were kept at room temperature without agitation or other treatment.

### 5.7 Nuclear Magnetic Resonance Spectroscopy

NMR spectroscopy experiments were conducted on a Bruker Avance spectrometer operating at 500 MHz and being equipped with a cryoprobe for increased sensitivity. Uranyl acetate was titrated to 92 μM monomeric ^15^N-labeled Aβ_40_ peptides in 20 mM MES buffer (90/10H_2_O/D_2_O) at either pH 7.3 or pH 5.1 at 5 °C. Two-dimensional ^1^H, ^15^N-HSQC (heteronuclear single quantum coherence) NMR spectra were recorded during the titrations using settings with 128 t1 increments, 24 scans, and a 1 s recycle delay. The spectra were then processed and evaluated in the Topspin software (v. 3.2) using already published assignments for HSQC cross-peaks of Aβ_40_ in buffer at neutral pH^157−159^ or at acidic pH.^119^

### 5.8 Binding Affinity Measurements via Tyrosine Fluorescence Quenching

The binding affinities between uranyl ions and Aβ_40_ peptides were evaluated via the quenching effect of uranyl on the intrinsic fluorescence of Tyr10, the only natural fluorophore in the wt Aβ peptide. Fluorescence measurements were conducted on a Jobin Yvon Horiba Fluorolog 3 fluorescence spectrophotometer (Longjumeau, France) using a quartz cuvette with a 4 mm path length. The fluorescence emission intensity of samples containing 20 μM Aβ peptides in 20 mM MES buffer, at either pH 7.3 or pH 5.5 and without or with 50 mM SDS detergent present, was measured at 305 nm (excitation wavelength 276 nm) at 20 °C. Aliquots of uranyl acetate (stock concentrations of 1, 2, and 10 mM) were titrated to the samples, and the Tyr10 fluorescence intensity was plotted against the concentration of UO_2_^2+^ ions. Apparent dissociation constants (*K*_D_^App^) were determined by fitting the plots to eq 2
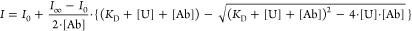
2where *I*_0_ is the initial fluorescence intensity with no added UO_2_^2+^ ions, *I*_∞_ is the steady-state intensity at the end of the titration, [Ab] is the protein concentration, and [U] is the concentration of added UO_2_^2+^ ions.^121,160^

### 5.9 Blue Native Polyacrylamide Gel Electrophoresis

Homogeneous solutions of oligomers of 80–100 μM Aβ_42_ peptides^132^ prepared (as described in Section 5.2) in the presence of different concentrations of uranyl acetate (0–1000 μM) were analyzed with BN-PAGE using the Invitrogen system (Thermo Fisher Scientific, USA). Thus, 4–16% Bis–Tris Novex gels (Thermo Fisher Scientific, USA) were loaded with 10 μL samples containing Aβ_42_ oligomer solutions alongside alongside the Amersham high MW calibration kit for native electrophoresis (GE Healthcare, USA). The gels were run at 4 °C using the electrophoresis system according to the Invitrogen instructions (Thermo Fisher Scientific, USA) and then stained with the Pierce Silver Staining Kit according to the manufacturer’s instructions (Thermo Fisher Scientific, USA). BN-PAGE was chosen for analysis instead of SDS–PAGE to avoid disruption of the SDS-stabilized and non-cross-linked Aβ_42_ oligomers by the high (>1%) SDS concentrations used in SDS–PAGE sample buffers.^161^

### 5.10 Infrared Spectroscopy

FTIR spectra of the SDS-stabilized Aβ_42_ oligomers (prepared as described in Section 5.2) were recorded in transmission mode on a Tensor 37 FTIR spectrometer (Bruker Optics, Germany) equipped with a sample shutter and a liquid nitrogen-cooled MCT detector. The unit was continuously purged with dry air during the measurements. 8–10 μL of the 80−100 μM Aβ_42_ oligomer samples, prepared (as described in Section 5.2) with different concentrations of uranyl acetate (0–1000 μM), was put between two flat CaF_2_ disks separated by a 50 μm plastic spacer covered with vacuum grease at the periphery. The assembled IR cuvette was mounted into the sample position of a sample shuttle inside the instrument’s sample chamber, while a metal grid (used as the background) was positioned in the reference holder. The sample shuttle was used to measure the sample and reference spectra without opening the chamber. The samples were allowed to sit for at least 15 min after closing the chamber lid to avoid interference from water vapor. FTIR spectra were recorded at room temperature in the 1900–800 cm^–1^ range, with 300 scans for both background and sample spectra, using a 6 mm aperture and at a resolution of 2 cm^–1^. The light intensities above 2200 cm^–1^ and below 1500 cm^–1^ were blocked with a germanium filter and a cellulose membrane, respectively.^162^ The spectra were analyzed and plotted with the OPUS 5.5 software, and second derivatives were calculated with a 17 cm^–1^ smoothing range.
